# Acid–base balance in hemodialysis patients in everyday practice

**DOI:** 10.1080/0886022X.2022.2094805

**Published:** 2022-07-06

**Authors:** Monika Wieliczko, Jolanta Małyszko

**Affiliations:** Department of Nephrology, Dialysis and Internal Disease, Medical University of Warsaw, Warsaw, Poland

**Keywords:** Acidosis, alkalosis, bicarbonates, hemodialysis

## Abstract

**Introduction:**

Abnormalities in blood bicarbonates (HCO_3_^–^) concentration are a common finding in patients with chronic kidney disease, especially at the end-stage renal failure. Initiating of hemodialysis does not completely solve this problem. The recommendations only formulate the target concentration of ≥22 mmol/L before hemodialysis but do not guide how to achieve it. The aim of the study was to assess the acid–base balance in everyday practice, the effect of hemodialysis session and possible correlations with clinical and biochemical parameters in stable hemodialysis patients.

**Material and methods:**

We enrolled 75 stable hemodialysis patients (mean age 65.5 years, 34 women), from a single Department of Nephrology. We assessed blood pressure, and acid–base balance parameters before and after mid-week hemodialysis session.

**Results:**

We found significant differences in pH, HCO_3_^–^ pCO_2_, lactate before and after HD session in whole group (*p* < 0.001; *p* < 0.001; *p* < 0.001; *p* = 0.001, respectively). Buffer bicarbonate concentration had only statistically significant effect on the bicarbonate concentration after dialysis (*p* < 0.001). Both pre-HD acid–base parameters and post-HD pH were independent from buffer bicarbonate content. We observed significant inverse correlations between change in the serum bicarbonates and only two parameters: pH and HCO_3_^–^ before hemodialysis (*p* = 0.013; *p* < 0.001, respectively).

**Conclusions:**

Despite the improvement in hemodialysis techniques, acid–base balance still remains a challenge. The individual selection of bicarbonate in bath, based on previous single tests, does not improve permanently the acid–base balance in the population of hemodialysis patients. New guidelines how to correct acid–base disorders in hemodialysis patients are needed to have less ‘acidotic’ patients before hemodialysis and less ‘alkalotic’ patients after the session.

## Introduction

Abnormalities in blood bicarbonates (HCO_3_^–^) concentration are a common problem in patients with chronic kidney disease, especially at the end-stage period, due to positive acid balance [1,2]. The body response to this is multifactorial and can lead to complications such as hypercatabolism, systemic inflammation, muscle wasting, reduced bone density, osteopenia/osteoporosis, increased fracture risk, cardiovascular, respiratory, immunological, and hormonal disturbances (e.g., insulin resistance) [2–5]. It also contributes to disease progression and increase in patient mortality [3,5,6]. The main source of acids in the body is proteins of animal origin consumed by patients (net production is ∼1 mEq/kg body mass/day) [2,7]. This production depends on the diet, but protein restriction in the diet in order to reduce acid production, leads to a negative nitrogen balance [2]. The substitution with vegetable protein carries the risk of hyperkalemia and should be used cautiously. Initiating of hemodialysis does not completely solve this problem and the questions with appropriate correcting metabolic acidosis have been a matter of debate for many years [8–15]. Hemodialysis patients require a supply of excess bicarbonate during each treatment to provide them with a relative acid–base balance between dialyses. According to American recommendations (K/DOQI-2000), the expected concentration of blood bicarbonate before mid-week hemodialysis treatment should not be lower than 22 mmol/L and a new K/DOQI recommendation (opinion) for nutrition (2020) indicate that it is reasonable to maintain serum bicarbonate levels between 24 and 26 mmol/L in patients in CKD 3–5 D [16,17]. The slightly more recent European recommendations (EBPG 2007) suggest blood bicarbonates before the same hemodialysis ought to be between 20 and 22 mmol/L [18]. The recommendations only recommend the expected titer but do not guide how to achieve them. At present, high HCO_3_^–^ dialysis fluids—mean 32–35 mmol/L (in USA 38–40 mmol/L)—are used to provide patients with sufficient bicarbonate for the period between hemodialysis [13,14]. High dialysate bicarbonates concentration and base administration can lead to rapid alkalization during hemodialysis and alkalosis after it and this one may cause arrhythmias resulting from hypokalemia, hypocalcemia or QT prolongation, vasodilation and hypotension, minute ventilation suppression, and accelerated vascular calcification [2,9,10,19,20]. Both, acidosis and alkalosis, are associated with an increased risk of mortality in patients receiving hemodialysis, and attention has been directed not only to serum bicarbonates but also to dialysate bicarbonate concentration itself [21]. In a large cohort study by DOPPS, a high dialysate buffer was associated with worse outcomes (all cause and cardiovascular hospitalizations) [22,23]. Some of the studies found that dialysate bicarbonate concentration had no effect on predialysis serum bicarbonates but it had a significant impact on the intra- and postdialysis alkalosis according to the elevated dialysate HCO_3_^–^ (30–32, 33–34, 35–36, and 37–40 mEq/L) [24,25]. It has been suggested that in addition to serum HCO_3_^–^, also pH should be measured. Thus, pH measurement is considered to be an important factor in determining morbidity and mortality in hemodialysis patients [8,13,14,26]. The aim of the study is an attempt to answer the question does individual selection of bicarbonate in bath, based on previous single tests, improve the acid–base balance in the population of hemodialysis patients in everyday practice—using the analysis of the effect of hemodialysis session and possible correlations with clinical and biochemical parameters in stable hemodialysis patients.

## Material and methods

The study enrolled 75 stable hemodialysis patients in a median age 67 and 7 years (34 women). Thirty-eight patients were older than 65 years. All Caucasian, treated in a single Department of Nephrology. For homogenicity of research, four patients treated with hemodiafiltration and two patients taking oral bicarbonates were not qualified to the study. All patients were on regular dialyses three times a week. All our patients qualified for the study were on a standard low-phosphate diet with protein content between 1 and 1.2 g/kg of body weight every day (median 1,17), we excluded patients with cachexia and neoplasm disease. Baseline parameters such as: duration of HD sessions, type of dialyzer, the dialyzer effective surface area, effective blood flow (Qb), dialysis fluid composition, and temperature, were recorded from the dialysis charts. Additionally, BMI, ultrafiltration volume, HCO_3_^–^ level in dialysate bath, type of vascular access, and heparin use (standard or low molecular weight heparins) were also recorded. Blood pressure and acid–base balance parameters were assessed on the same day—before and after mid-week hemodialysis session. Standard dialysate bath contained 32 mmol/L of HCO_3_^–^ and 3 mmol/L of acetate, giving a final product of 35 mmol/L base delivered. The HCO_3_^–^ content of the dialysate used for dialysis fluctuated in the range from 28 to 36 mmol/L. The decisions about HCO_3_^–^ in the bath were made by the clinician in relation to previous laboratory tests. The other dialysate components were Na = 138 mmol/L, K = 2–4 mmol/L, Ca = 1.25 or 1.5 mmol/L, Cl = 110 mmol/L, Mg = 0.5 mmol/L, glucose = 1 g/L. Predialysis and postdialysis acid–base status was measured immediately in the central laboratory using standard methods. Statistical analysis was carried out based on the Statistica Program (13.3.721.1 pl 64 bit license: The Medical University of Warsaw). Student’s t-test and Pearson analysis were used.

## Results

Clinical characteristic of the patients and hemodialysis parameters are presented in Table 1. There were no differences in pH and HCO_3_ between groups women and men before and after HD and no differences between patients older and younger than 65 years. The residual kidney function was found only in 7 patients (9,3%) (median 0,5 L per day), and 6 of them were taking diuretics.

**Table 1.** Baseline clinical characteristics and hemodialysis parameters.

Parameters	Overall (*n* = 75) Mean ± SD	Range	Reference values	Median
Age (years) > 65 age (%)	65.5 ± 15.8 50.7	25.8-91.4		67.17
Gender (F/M)	34/41 (45% F)			
Diabetes	27 (36%)			
BMI (kg/m²)	26.15 ± 6.01	12.34-44.75		24.54
CKD time > CKD stage 1 (years)	12 ± 10	1.5 month-45 years		10
Dialysis treatment time (years)	4.3 ± 10.3	1.5 month −25 years		2.17
A-V fistula/catheter	48/27 (64% fistula)			
Kt/V	1.35 ± 0.18	0.66–1.67	>1.4	1.4
Hemoglobin (g/dL)	10.57 ± 1.34	6.9–14.2	10.5–11.5	10
Albumin (g/dL)	4.12 ± 0.38	3–4.9	3.5–4.5	4.2
Hemodialysis duration (hours)	4.065 ± 0.427	3–5		4.0
Bicarbonates in dialysate (mmol/L)	32.92 ± 1.78	28–36		32
UF (mL)	2017 ± 904	100–3700		2000
Qb (mL/min)	267 ± 27	200–350		250
Effective surface area (m^2^)	1.511 ± 0.3078	1–2.1		1.4

### Hemodialysis parameters

A median of Kt/V was 1.4, single session HD duration time = 4.0 h, effective surface area = 1.4 m^2^. A median of effective blood flow (Qb) was 250 mL/min, dialysate flow (Qd) was 500 mL/min (standard in Poland), median BMI = 24.54, ultrafiltration volume = 2000 mL, HCO_3_^–^ in dialysate bath = 32.0 mmol/L. The volume of UF depends on patient’s body weight, residual renal function, and symptoms of overhydration, but we try to make it no more than 2% of dry weight-UF median in our study was 2000 mL and the volume of the ultrafiltration correlated with body weight (*p* = 0.06) but not with pH and bicarbonates pre- and post-HD.

Eight patients were dialyzed by using high-flux dialyzers. There were no differences in pH and HCO_3_ between the two separate groups dialyzed by using high-flux and low-flux dialyzer before and after HD. There were no correlations between pH, HCO3 and HD time, effective surface areas, Qb, and ultrafiltration volume in whole group.

### Vascular access and heparin use

Most of the patients were hemodialyzed by using arteriovenous fistula (48 patients − 64%) and received low molecular weight heparins (46 patients − 61,3%). In 27 patients (36%) dialyzed through a central venous catheter (CVC), we found their predialysis and postdialysis pH significantly lower than in those with a native arteriovenous fistula (pH pre-HD = 7.307 vs. 7.371 *p* < 0.001; pH post-HD = 7.363 vs. 7.439, *p* < 0.001), while, what’s interesting, predialysis and postdialysis HCO_3_^−^ levels did not differ between both group (HCO_3_^–^ pre-HD 21.64 vs. 21.98, *p* = ns; HCO_3_^–^ post-HD 27.156 vs. 27.213, *p* = ns) even though there were not significant differences in the utrafiltration volume, effective blood flow, bicarbonate content in dialysate bath, HD duration between groups (Table 2).

**Table 2.** Comparison of pre-vs. post-HD acid–base parameters in two group: dialyzed *via* arterio-venous fistula (A-V fistula) and CVC.

	A-V fistula	CVC	*p*
pH pre-HD	7.371	7.307	<0.001
HCO3– pre-HD	21.98	21.64	NS
pH post-HD	7.439	7.363	<0.001
HCO3– post-HD	27.213	27.156	NS

What’s also interesting, in the group treated with low molecular weight heparins, we have noticed significantly higher serum postdialysis bicarbonate level (*p* = 0.03).

### Acid–base blood parameters

A median of blood parameters before HD session were: pH = 7.357, pCO_2_ 40.4 mmHg_,_ HCO_3_^–^ 21.7 mmol/L, a median of change bicarbonates during hemodialysis was 5,3 mmol/L; post-HD: pH 7.417, pCO_2_ 42.6 mmHg, HCO_3_^–^ 27.2 mmol/L.

Post-HD pH and HCO_3_^–^ correlated with pre-HD pH and HCO_3_^–^, respectively, and only post-HD HCO_3_^–^ correlated significantly with a bicarbonate concentration in dialysate. Other correlations pre- and post-HD serum HCO_3_^–^ and pH value are presented in a Table 3. There were not significant correlations in the ultrafiltration volume, hemoglobin, effective blood flow, bicarbonate content in dialysate bath, and HD duration between groups.

**Table 3.** pH and serum bicarbonate prehemodialysis and posthemodialysis—significant correlations (UF – ultrafiltration volume, Qb – blood flow).

	pH pre- HD	HCO_3_^–^ pre- HD	pH post- HD	HCO_3_^–^ post- HD	Δ HCO_3_^–^	Hb pre-HD	Albumin pre-HD	UF	HD duration	Qb	HCO_3_^–^ bath
pH pre-HD		0.3354 *p* = 0.003	0.6979 *p* < 0.001	NS	−0.2866 *p* = 0.013	NS	NS	NS	NS	NS	NS
HCO3– pre-HD	0.3354 *p* = 0.003		NS	0.5630 *p* < 0.001	−0.6448 *p* < 0.001	NS	NS	NS	NS	NS	NS
pH post-HD	0.6979 *p* < 0.001	NS		NS	NS	NS	0.3265 *p* = 0.004	NS	NS	NS	NS
HCO3– post-HD	NS	0.5630 *p* < 0.001	NS		NS	NS	0.3175 *p* = 0.06	NS	NS	NS	0.4892 *p* < 0.001
ΔHCO3–	–0.2866 *p* = 0.013	−0.6448 *p* < 0.001	NS	NS		NS	NS	NS	NS	NS	NS
HCO3– bath	NS	NS	NS	0.4892 *p* < 0.001	NS	NS	0.2909 *p* = 0.011	NS	NS	NS	

We have found significant differences in pH, HCO_3_^–^ pCO_2_, and lactate changes before and after HD session in the whole studied group (Table 4). Buffer bicarbonate concentration had only statistically significant effect on the bicarbonate concentration after dialysis. Both pre-HD acid–base parameters and post-HD pH were unrelated to buffer bicarbonate content. Significant inverse correlations between change in the serum bicarbonates and two parameters: HCO_3_^–^ and pH before hemodialysis were found.

**Table 4.** Statistically significant changes in the acid–base and urea/lactate balance during dialysis.

Parameter	Before hemodialysis	After hemodialysis	*p* value
pH	7.348 ± 0.057	7.412 ± 0.053	<0.001
Bicarbonates (mmol/L)	21.86 ± 2.38	27.19 ± 1.79	0.00
pCO2 (mmHg)	41.07 ± 5.78	44.02 ± 6.8	<0.001
Lactates (mmol/L)	1.71 ± 0.68	1.44 ± 0.57	<0.01
Urea (mg/dL)	128.89 ± 33.26	42.76 ± 18.01	0.00

### HCO_3_^–^ in dialysate bath

Standard dialysate, with a total base content of 35 mmol/L was used in only 31 cases (41.3%). In other patients, different total base dialysate was used: 31 mmol/L in 2 cases (2.7%), 33 mmol/L in 6 cases, (8%) 36 mmol/L in 6 cases (8%), 37 mmol/L 11 cases (14.7%), 38 mmol/L 18 cases (24%), and 39 mmol/L in 2 cases (2.7%), which was in line with previous monthly tests.

### Serum HCO_3_^–^ before HD

In the whole group, there were 38 patients (50.7%) with serum bicarbonate < 22 mmol/L before hemodialysis and 13 patients (17.3%)<20 mmol/L; pH value was in acidotic range (<7.35) in 36 pts (48%).

Using a standard HCO_3_^–^—based dialysate with a total base content of 35 mmol/L—only 15 patients (48%) had the recommended minimum 22 mmol/L HCO_3_^–^ before hemodialysis, while 16 pts (51.6%) had predialysis HCO_3_^–^<22 mmol/L, and 3 pts (9.7%) even < 20 mmol/L. In this group, 12 patients (38.7%) presented pH values within the normal range (7.35–7.45), while, as many as 18 patients (58.1%) presented predialysis pH < 7.35, and minimal pH value was 7.209. One patient had alkalosis before hemodialysis (1,33%). The person with alkalotic pH before HD had one and half liters per day diuresis and she was not taking diuretics. Thus, no correlation was found between the amount of urine output, diuretics, and the acid–base parameters before and after HD. No differences were found between groups with and without urine output.

No significant differences were found between groups pre-HCO_3_ < and > 22 mmol/L, except for a few the acid–base parameters: before HD (pH, HCO_3_, and pCO_2_) and post HD (HCO_3_ and pCO_2_). In patients group with HCO3 < 22mmol/L we found only a few significant correlations: pre-HD potassium with pre-HD phosphorus (*r* = 0,53), body weight and height (*r* = –0,34 and −0,38 respectively), pre-HD phosphorus with pre- and post-HD: potassium, lactate, and urea (*r* = 0,53; 0,33; −0,34; −0,35; 0,34; 0,34, respectively); neither the concentration potassium nor phosphorus did not correlate with acid–base parameters, albumin, hemoglobin, protein intake, length of hemodialysis, and volume of ultrafiltration.

The comparison of predialysis acid–base parameters depending on the used dialysate is presented in Table 5.

**Table 5.** Comparison of pre-dialysis acid–base value in all dialysis patients depending on the used dialysate (in red color values outside the recommendation).

HCO3^–^ bath	Total base	No of pts	pH mean	HCO3^–^ mean	p*H* < 7.35 (pts/%)	pH 7.35–7.45 (pts)/%	p*H* > 7.45 (pts)	HCO3^–^ <20 mmol/L (pts)	HCO3^–^ <22 mmol/(pts)	HCO3^–^ >22 mmol/L (pts)	HCO3^–^ >26 mmol/L (pts)
28	31	2	7.355	19.15	2/100	0	0	1	1	1	0
30	33	6	7.349	20.43	3/50	3/50	0	2	3	1	0
32	35	31	7.336	22.06	18/58	12/38.7	1	3	16	15	2
33	36	5	7.366	22.16	2/40	3/60	0	1	4	0	1
34	37	11	7.336	21.4	7/63.6	4/36.4	0	3	7	4	0
35	38	18	7.379	22.63	3/16.7	15/83.3	0	1	8	10	2
36	39	2	7.267	20.6	1/50	1/50	0	2	2	0	0

### Serum HCO_3_^–^ after HD

In the whole group, we found 62 patients (82.7%) with HCO_3_^–^>26 mmol/L directly after HD, 24 patients (29.3%) with HCO_3_^–^>28 mmol/L, and 4 patients (5.3%) with HCO_3_^–^≥30 mmol/L; 21 patients (28%) had pH in alkaloid range (>7.45), but 11 patients (14.7%) were still acidotic. All acidotic patients were dialyzed using total base <38 mmol/L and most of them received bicarbonates from a standard dialysate (with total base 35 mmol/L). The change in bicarbonates during HD was 5.45 ± 1.99 mmol/L (range 0.4–10.4, median 5.3 mmol/L). The patients who were still acidotic post-HD were well dialyzed and well nourished: the median kT/V in this group was 1,4, the median albumin was 3,9 g/dL and their median protein intake was no different to other patients.

Using a standard HCO_3_^–^—based dialysate with a total base content of 35 mmol/L—after hemodialysis 26 patients (83.9%) had HCO_3_^–^>26 mmol/L, no patient had HCO_3_^–^<22 mmol/L, and a minimal HCO_3_^–^ was 22.6 mmol/L; 4 pts (12.9%) had pH > 7.45, while 21 patients (67.7%) had pH in normal range; as many as 6 patients (19.4%) have remained acidotic (pH < 7.35).

**Figure 1.**
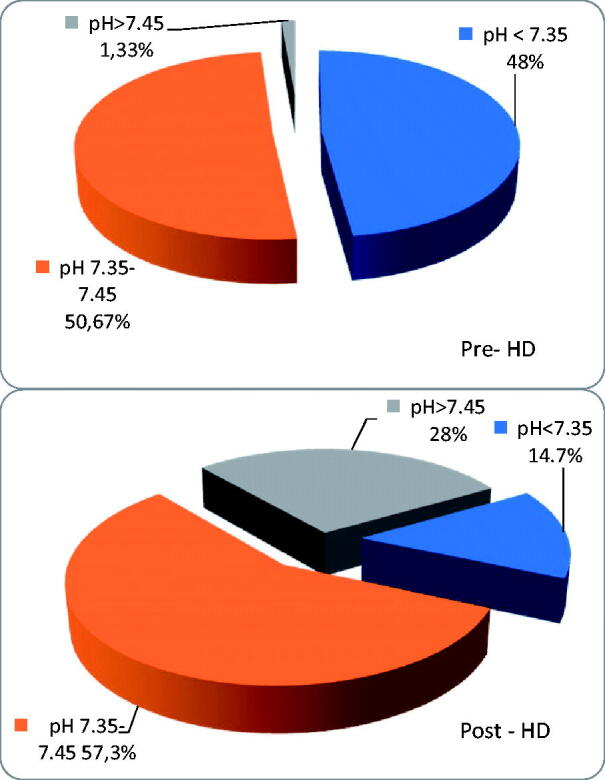
Pre-and post-HD pH values.

**Figure 2.**
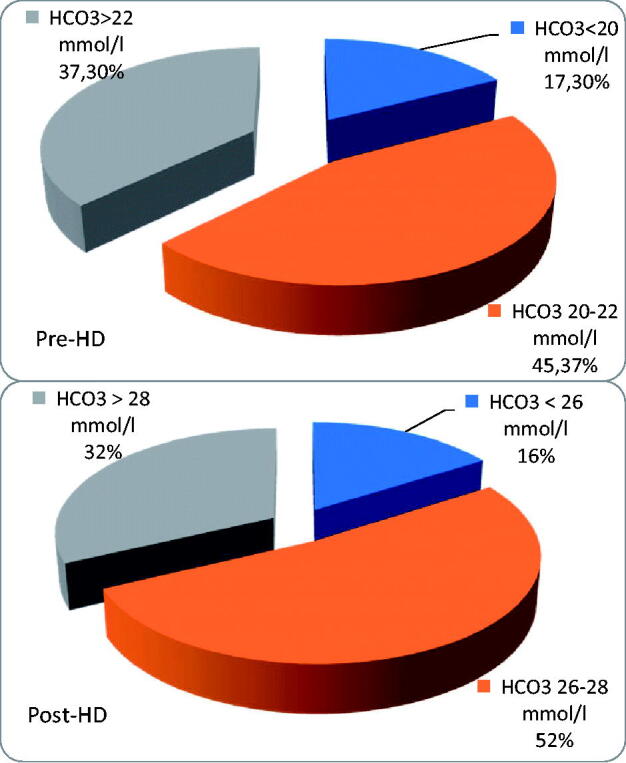
Pre-and post-HD serum HCO_3_^–^.

The distributions of prehemodialysis and posthemodialysis pH and pre-HD and post-HD HCO_3_^–^ are shown in Tables 5 and 6 and Figures 1 and 2.

**Table 6.** Comparison of postdialysis acid–base value in all dialysis patients depending on the used dialysate (in red color values outside the norm).

HCO3^–^ bath	Total base	No of pts	pH mean	HCO3^–^ mean	p*H* < 7.35 (pts)/%	pH 7.35–7.45 (pts)/%	p*H* > 7.45 (pts)/%	HCO3^–^ <26 mmol/L (pts)	HCO3^–^ 26–28 mmol/(pts)	HCO3^–^ 28–30 mmol/L (pts)	HCO3^–^ >30 mmol/L (pts)
28	31	2	7.427	23.75	1/50	1/50	0	0	0	1	1
30	33	6	7.421	25.98	1/16.7	3/50	2/33.3	2	3	1	0
32	35	31	7.393	26.95	6/19.4	21/67.7	4/12.9	5	19	6	1
33	36	5	7.418	26.34	1/20	2/40	2/40	2	3	0	0
34	37	11	7.412	27.69	2/18.2	6/54.5	3/27.3	0	6	5	0
35	38	18	7.446	28.04	0	9/50	9/50	1	8	9	0
36	39	2	7.326	29.8	0	1/50	1/50	2	0	0	0

## Discussion

Our study presents the real-world data in university hospital dialysis unit. In our study, we found many patients who were ‘acidotic’ before hemodialysis and ‘alkalotic’ after HD. Even though the mean HCO_3_^–^ in our cohort (21.86 ± 2.3807 mmol/L) was similar to DOPPS study from 1996 to 2001, the number of patients who had bicarbonate < 21 mmol/L before hemodialysis (like in analysis) turned out to be a little lower and was 33.3 vs. 35% (25 patients) [23,27]. In the study by Vashistha et al. [28] on patients dialyzed in DaVita facilities in 2001–2006, still 40% hemodialysis patients had average predialysis serum bicarbonate < 22 mmol/L and only 5% ≥27 mmol/L. In the study by Bozikas *et al.* [20], 70% of the patients had serum bicarbonate levels below 22 mmol/L as recommended by the K/DOOQI guidelines, and 30% have their postdialysis pH in the ‘alkalotic’ range. In our patient’s group, serum predialysis bicarbonates were as follows: 50.7% of patients had bicarbonate levels < 22 mmol/L, 1.33% patients ≥ 27 mmol/L, and 5.33% patients ≥ 26 mmol/L, and 1,33% patients had pH in alkalotic range. In our studied population more patients were acidotic and fewer alkalotic in comparison with the DaVita population [28] and fewer acidotic patients than reported by Bozikas *et al.* [20]. As shown by Gennari [21] in the review article, pre-HD and post-HD bicarbonate levels were highly variable with the average serum predialysis bicarbonate ranging from 17 mmol/L to above 27 mmol/L, and post HD serum bicarbonate being 4–7 mmol/L lower than the dialysate bicarbonate level. In our study, the serum postdialysis bicarbonates were 4.02–6.96 mmol/L lower the dialysate HCO_3_^–^ level.

Not surprisingly, significant changes in pH, HCO_3_^–^ pCO_2_, lactate changes during HD were found in our study. In patients who are dialyzed thrice weekly, standard HD (4 h) caused large fluctuations of serum bicarbonates during each session which were not physiological. The net bicarbonate change was the greatest when the concentration of HCO_3_^–^ in used dialysate was the lowest (9.2 ± 0.27) and the slightest when the concentration of HCO_3_^–^ in used dialysate was the highest (4.6 ± 0.28). Using the standard dialysate (35 mmol/L) the bicarbonate change was mean 4.96 ± 1.9. We found strong inverse correlations between the change of bicarbonates and only two parameters: HCO_3_^–^ and pH before hemodialysis. A similar observation was made by Uribarri *et al.* [29], Sepandj *et al.* [30], and Noh *et al.* [31], but the number of patients in their studies were lower (15, 70, and 53, respectively). The fluctuations were the largest in the most acidotic patients.

Similarly to some other studies [24], we found that the dialysate bicarbonates concentration had no effect on predialysis serum bicarbonate, and in our study group the postdialysis serum bicarbonates were related to the elevated dialysate HCO_3_^–,^ even though the mean pH was within the normal range.

In the previous studies, negative correlations between the change in serum bicarbonate during the HD and the bicarbonates predialysis level were found, so patients with the highest bicarbonates level at the initiation of the treatment experienced its modest increase [1,30,31]. In our cohort, the change in serum bicarbonate during HD significantly negatively correlated with predialysis serum bicarbonate concentration.

It is known that both high- and low-serum bicarbonate levels have been associated with an increased risk of mortality [23,28,32,33] and serum bicarbonate were inversely related to serum albumin and phosphate levels in patients with end stage renal disease [34]. In our cohort, only the level of serum postdialysis bicarbonate correlated with serum albumin, but it was not related to serum phosphate level.

The previous studies have shown that Americans aged 65 and over have higher serum bicarbonate levels than younger Americans, partly due to lower animal protein intake and hence lower dietary acid load. In our study, there were 38 patients older than 65 age, (50.7%) and 37 pts (49.3%) below this age. Older age was also associated with higher serum bicarbonate level in patients with end stage renal disease [28,34,35]. In our study, there were no differences in serum bicarbonate in relation to age.

In our cohort, only 27 patients (36%) were dialyzed using a CVC and their predialysis and postdialysis pH values were significantly lower than those with a native arteriovenous fistula, while predialysis and postdialysis HCO_3_^–^ levels did not differ between both the groups. This observation is similar to study by Bozikas *et al.* [20] who found the same differences in their study group, which had 1/3 patients dialyzed through a CVC.

Fülöp et al. [36] encountered worse tolerance of higher HCO3 on on-line HDF, rather than plain HD suggesting a more propound HCO3 influx during on-line hemodiafiltration and the concerns described here are like more profound during on-line HDF. Leypoldt *et al.* [37] recently described mathematical model can predict the effect of operating conditions on acid–base balance within and exiting the extracorporeal circuit during continuous kidney replacement therapy.

Despite the improvement in hemodialysis techniques, acid–base balance still remains a challenge, there is a huge discrepancy between recommendation publishes several years ago and real-world data. New guidelines how to correct acid–base disorders in hemodialysis patients are needed to have less ‘acidotic’ patients before hemodialysis and less ‘alkalotic’ patients after the session. Currently, no studies are available addressing this issue. It appears that individualization of the standard HD session would be of value, however, we need more large-scale studies to prove or disprove this hypothesis [11]. A change in dialysate content or a bicarbonate profiling could also be considered as an option. Sodium bicarbonate supplementation therapy could also be taken into account as reported Kourtellidou *et al.* in randomized controlled trial [38]. Although alkali treatment (sodium citrate or sodium bicarbonate) would be helpful, we should remember that this type of intervention is not free from side effects. Sodium citrate can lead to increase gastric aluminum absorption and sodium bicarbonate—to bloating and flatulence [39–41].

Maybe we are using wrong assumptions? Maybe lowering the bicarbonate content in the dialysate will help to better correct the acidosis by better exhaling the carbon dioxide, as reported Cove *et al.* and Montagud-Marriani *et al.* Sargent *et al.* also reported that reducing bath HCO_3_^–^ should decrease organic acid production [42,43].

We are fully aware of the limitations of our study, first if all being single center. Although more patients were enrolled in our study than in other studies, the group is still could be larger. Another limitation is the observational nature of the study; however, our assumption was to analyze parameters in real life.

## Conclusions

Despite the improvement in hemodialysis techniques, acid–base balance still remains a challenge. Our study has shown that the selection of bicarbonate level in the hemodialysis bath based on previous periodic laboratory test does not results in a permanent improvement of the acid–base balance. We need prospective randomized multicenter studies to have more important information to formulate a new guideline. We need long-term analysis and, we believe, mathematical modeling to improve and individualize the acid–base balance in hemodialysis patients.
